# The Influence of Innate Lymphoid Cells and Unconventional T Cells in Chronic Inflammatory Lung Disease

**DOI:** 10.3389/fimmu.2019.01597

**Published:** 2019-07-11

**Authors:** Jessica G. Borger, Maverick Lau, Margaret L. Hibbs

**Affiliations:** ^1^Department of Immunology and Pathology, Central Clinical School, Monash University, Melbourne, VIC, Australia; ^2^Department of Pharmacology and Therapeutics, Lung Health Research Centre, University of Melbourne, Melbourne, VIC, Australia

**Keywords:** ILC, MAIT, NK cell, NKT cells, γδ-T cell, lung, chronic lung disease

## Abstract

The lungs are continuously subjected to environmental insults making them susceptible to infection and injury. They are protected by the respiratory epithelium, which not only serves as a physical barrier but also a reactive one that can release cytokines, chemokines, and other defense proteins in response to danger signals, and can undergo conversion to protective mucus-producing goblet cells. The lungs are also guarded by a complex network of highly specialized immune cells and their mediators to support tissue homeostasis and resolve integrity deviation. This review focuses on specialized innate-like lymphocytes present in the lung that act as key sensors of lung insults and direct the pulmonary immune response. Included amongst these tissue-resident lymphocytes are innate lymphoid cells (ILCs), which are classified into five distinct subsets (natural killer, ILC1, ILC2, ILC3, lymphoid tissue-inducer cells), and unconventional T cells including natural killer T (NKT) cells, mucosal-associated invariant T (MAIT) cells, and γδ-T cells. While ILCs and unconventional T cells together comprise only a small proportion of the total immune cells in the lung, they have been found to promote lung homeostasis and are emerging as contributors to a variety of chronic lung diseases including pulmonary fibrosis, allergic airway inflammation, and chronic obstructive pulmonary disease (COPD). A particularly intriguing trait of ILCs that has recently emerged is their plasticity and ability to alter their gene expression profiles and adapt their function in response to environmental cues. The malleable nature of these cells may aid in rapid responses to pathogen but may also have downstream pathological consequences. The role of ILC2s in Th2 allergic airway responses is becoming apparent but the contribution of other ILCs and unconventional T cells during chronic lung inflammation is poorly described. This review presents an overview of our current understanding of the involvement of ILCs and unconventional T cells in chronic pulmonary diseases.

## Introduction

The lungs are constantly exposed to particulates from the environment. This inhaled matter includes harmless aeroallergens, airborne pathogens which can cause infection, and noxious agents including dust, smoke, and other environmental pollutants that can induce lung tissue damage. The conducting airways of the respiratory system are comprised of ciliated epithelium interspersed with mucus-producing goblet cells. This protects against particulates and infectious agents, which adhere to the mucus, and through the actions of the cilia, are cleared from the airways. This barrier is also supported by the presence of tissue-resident immune cells that provide cellular and humoral host defense. The resting lung harbors a plethora of leukocytes including B and T cells and myeloid cells (alveolar macrophages, interstitial macrophages, monocytes, dendritic cell subsets, neutrophils, eosinophils, mast cells), with their numbers and proportions changing dramatically during infection and inflammation. In response to tissue damage, stress or infection, the respiratory epithelium secretes proteins important in inflammation and host defense such as surfactant, anti-microbial peptides (β-defensins, cathelicidins), danger signals such as alarmins, chemokines, and cytokines to recruit and activate immune cells. While this normally results in the clearance of the pathogenic agent, in certain situations the inflammatory response becomes chronic leading to progressive lung tissue damage. Defining the cellular and molecular pathways that are altered during chronic disease transition may reveal new lung disease targets for therapy.

Recent studies have revealed the presence of small populations of other distinct immune cells in the lung including innate lymphoid cells (ILCs) and unconventional T lymphocytes (CD1-restricted NKT, MAIT cells, and γδ-T cells) and described their contribution to lung homeostasis, however these innate-like cells are now also emerging as pathogenic mediators in lung disease. ILCs show transcriptional and functional parallels to the conventional polarized T helper cell subsets (Th1, Th2, Th17), with the critical difference that ILCs lack clonally distributed antigen-specific receptors, responding instead to danger and stress signals derived from mucosal epithelium, stroma, and myeloid-lineage cells. In contrast, γδ-T cells, MAIT cells, and NKT cells possess semi-variant antigen-specific receptors but likewise respond to similar mediators, although these are more restricted dependent on cell type, and in some cases, are still poorly defined. Here we will summarize how these relatively new players participate in immune-regulation and homeostasis in the lung, and elaborate on how they can become dysregulated and contribute to chronic lung diseases.

## ILCs in Lung Homeostasis

**Figure 1:**
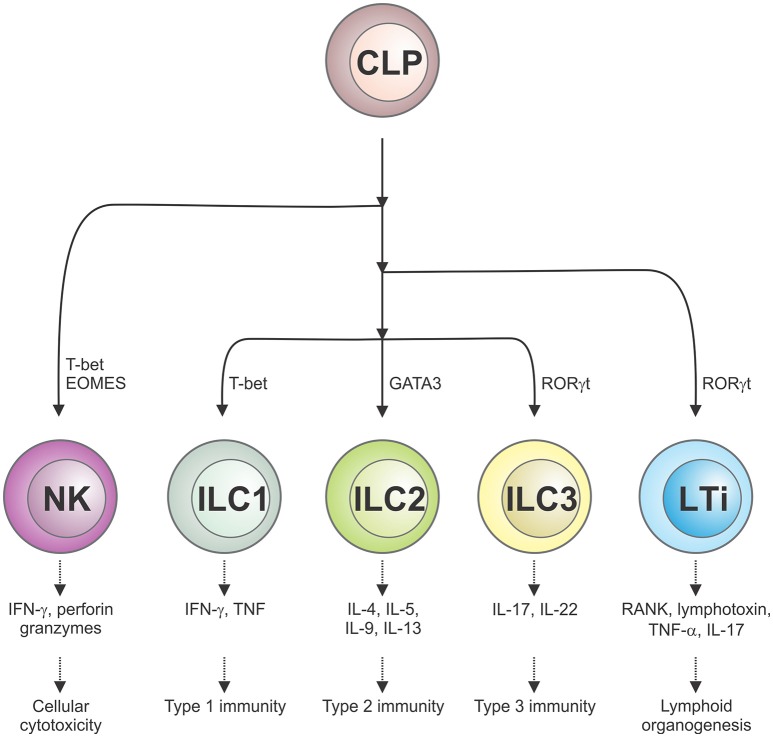
Schematic representation of the five different ILC subsets and their roles in cellular immunity. ILCs, which are derived from a common lymphoid progenitor (CLP), are classified into five distinct subsets on the basis of their transcription factor usage and unique spectrum of cytokines that they produce. This confers their distinct roles in immune responses and lymphoid organ development.

The ILC family is comprised of several phenotypically distinct subsets that are derived from a common lymphoid precursor, which unlike conventional T lymphocytes, do not express antigen-specific receptors nor mediate antigen-specific immune responses (1). ILCs are classified into five distinct subsets based on their development, transcription factor expression and effector function (2). Conventional NK cells, which are dedicated cytotoxic effectors that kill virus-infected cells and tumor cells and require the transcription factor T-bet for their function, are now regarded as a distinct ILC subset. A second subset, lymphoid tissue-inducer (LTi) cells, are responsible for secondary lymphoid organogenesis, while the remaining three subsets of ILCs (ILC1, ILC2, ILC3) play a role akin to helper T cells. Herein, we will use the abbreviation ILCs to refer to the ILC1, 2, and 3 subsets. Much like Th1, Th2, and Th17 cells, ILC1 express T-bet (encoding for their cytotoxic potential), ILC2 express GATA-3 while CD4^−^ ILC3 and CD4^+^ LTi cells express retinoic acid-related orphan receptor (ROR)γt (Figure 1). Although distributed in both lymphoid and non-lymphoid organs throughout the body, ILCs populate barrier surfaces, notably the skin, lung, and other mucosal sites, in much greater numbers.

Under homeostatic conditions, the immune cell make-up of the mouse lung is predominantly conventional lymphocytes and myeloid cells, although NK cells and all three ILC subsets have also been identified in the airways, albeit at much lower frequencies (3, 4). The ILC2 population is thought to be tissue-resident and thus are the predominant helper-type ILCs in the steady-state mouse lung (3, 5). Indeed, in a parabiotic experiment, ILC2s found in the lung were shown to be of host origin and strikingly, very few ILC2s were found in the circulation with the vast majority residing within the lung parenchyma (6). Furthermore, during homeostatic conditions, self-renewal of ILC2s maintained the tissue-resident population (6). Even during acute infection with the helminth *Nippostrongylus brasiliensis*, the ILC2 population in the lung remained host-derived and it was not until the expulsion of the larvae, which is associated with inflammation and tissue repair, that an increase in donor-derived ILC2s was observed (6). Similarly, in another parabiosis study, after 1 month of shared circulation, T cells and eosinophils were present from both host and donor, while ILC2s of the parabiotic donor could not be detected, even 1 week after intra-tracheal IL-33 challenge (7).

Unconventional T cells are similarly thought to be tissue-resident and arise from local expansion. Indeed, γδ-T cells, with a restricted Vγ4 or Vγ6 T cell receptor (TCR), mature in the thymus as CD44^+^CD27^−^ with a defined effector phenotype of IL-17 production (γδ-T17 cells) and specifically populate lung tissue during fetal development (8). CCR6/CCR2 co-expression identifies tissue-residency of γδ-T cells including γδ-T17 cells, but a recent study has identified populations of γδ-T17 cells lacking CCR6 expression in lung tissue (9) suggesting that under specific conditions, other γδ-T cell subsets are able to migrate and populate the lungs. Evidence now suggests that inflammatory ILC2s from the intestine are also able to migrate to the lung under specific conditions (10, 11). As both the lung and gut associated tissues exhibit tonic immune activation due to interactions between the mucosa and microbiota, further evidence is required to understand the tissue and context-specific regulation of ILC and unconventional T cell responses.

Maintenance of the epithelial barrier of the respiratory tract is critical to limit exposure to pathological and immunological stimuli and ILC2s are thought to have a key role in this process. ILC2 mediators including IL-4, IL-5, IL-9, IL-13, and the epidermal growth factor-like molecule amphiregulin are critical for maintaining airway barrier integrity and tissue homeostasis. In the lung, ILCs have been shown to interact with local epithelial cells and myeloid cells. ILC2 depletion impaired airway epithelial barrier integrity following influenza virus infection through a failure to generate hyper-plastic epithelial cells, an epithelial repair response, causing epithelial cells to undergo necrosis leading to deterioration of the epithelial lining (5). To modulate epithelial cell responses in the airways, ILC2s may use autocrine IL-9 to promote IL-5 and IL-13 production. Exposure to chitin, which is associated with allergic responses, was shown to drive IL-33 and thymic stromal lymphopoietin (TSLP) from resident alveolar type II cells, inducing the IRF4-IL-9 module in ILC2s, highlighting a critical interaction between resident ILCs and structural cells (12). Moreover, IL-5 and IL-13 have been shown to promote mucus hyper-production and tissue repair in type 2 immune responses such as asthma and helminth infection [reviewed in (13)]. Interestingly in the human lung under homeostatic conditions, it was reported that ILC3s are the most abundant ILC population in healthy older individuals, although in this study the confounding effects of smoking (two out of five subjects sampled) and elevated BMI necessitates further investigation (14). In the lung, IL-22 is mainly produced by ILC3, in addition to γδ-T cells, and Th17 cells, and has also been shown to be involved in the maintenance of epithelial barrier function, mucus production and tissue repair [reviewed in (15)]. Thus, ILCs contribute to barrier surveillance and epithelial protection and repair through coordinated interactions with other cells in the lung.

Epithelial cell-derived mediators are critical in the regulation of ILC responses. Epithelial cell or myeloid cell derived IL-25, IL-33, and TSLP can promote an ILC2 response after allergen challenge, helminth exposure or influenza infection. IL-33, an alarmin that is highly expressed in airway epithelial cells, has been shown to be overexpressed in lungs of patients with pulmonary fibrosis, asthma, and COPD and has been shown to be a main driver of ILC2 expansion (16–18). In mice following challenge with influenza virus, release of IL-33 corresponded with elevated IL-5 production by ILC2s and the recruitment of eosinophils to the lung during the recovery phase of influenza infection (19).

Intriguingly, IL-17 production from γδ-T cells was shown during neonatal influenza to augment IL-33 production from the mucosa, generating a type 2 response and the production of amphiregulin by ILC2s (20). The authors found a similar correlation of IL-17, IL-33, and amphiregulin in the nasal wash of human infants infected with influenza, identifying an axis of IL-17 production from γδ-T cells in the production of IL-33 for ILC2 repair responses in the lung (20).

ILC2 cells also express the receptor for TSLP, and together with IL-25 and IL-33, TSLP has been shown to induce ILC2s following rhinovirus and respiratory syncytial virus infection (21–23). A suggestive association with a SNP in the TSLP gene and severe asthma has been identified in genome-wide analysis of severe asthmatics in one study (24), and TSLP has been shown in a papain-model of asthma to illicit a type 2 response for which ILC2s were the dominant source of IL-13 and IL-5 (25). Similarly, TSLP has been shown to play an important role in iNKT cell-dependent asthma, enhancing AHR expression by increasing iNKT cell production of IL-13 (26). TGF-β, also secreted by lung epithelial cells, has been shown to skew ILC responses causing the upregulation of the TGF-β receptor (TGF-βRII) and enhancement of ILC2 activity, demonstrating that ILCs take direct cues from the lung mucosa and epithelium (27). This would suggest that the particular location of ILCs within the lung and the involvement of unconventional T cells is responsible for directing the nature and magnitude of the specific immune response.

ILCs themselves have been reported to antagonize each other, with IL-27 and interferon-γ (IFN-γ) released by ILC1s shown to inhibit ILC2s and type 2 responses in the lung (28). In addition and more fully discussed below, ILCs are highly plastic, being able to readily change phenotype and function depending on their microenvironment. The contribution of other innate-like cells such as γδ-T cells, MAIT cells, and NKT cells in directing ILC traits and functionality in the lung may be of great importance in understanding the role of ILCs in chronic lung disease, in particular when compounded by viral, bacterial, and other disease exacerbations.

## Plasticity of ILCs During Inflammation

Cellular plasticity is the ability of a differentiated effector cell to adapt their function in response to altered situations and is a particular feature of CD4^+^ T cells (29). Emerging evidence indicates that ILC subsets also demonstrate considerable plasticity in response to the inflammatory milieu as well as viral and bacterial challenge and tissue site residency. This is potentially due to the promiscuous co-expression of multiple transcription factors driving differential cytokine expression as well as an ability to alter cell surface marker expression in the face of a changing environment.

## ILC1 and NK Cell Plasticity

Historically, NK cells were categorized as cytolytic ILC1s but more recently have been identified as one of the five bona fide ILC populations (2). At steady state, ILC1 and NK cells can be identified by differential expression of TRAIL, CD49a, and CXCR6 and the transcription factors Eomes and promyelocytic leukemia zinc finger protein (PLZF), however during inflammation these surface markers and transcription factors can become altered which impedes the ability to track bona fide ILC1s. The cytokine milieu is largely responsible for diverting ILC identity as has been shown for ILC2s and ILC3s, which are capable of expressing T-bet and other T-bet related gene signatures as well as secreting IFN-γ. Although NK cells in the bone marrow are critically reliant on T-bet, mature cell fate is regulated by additional expression of Eomes. Indeed, deletion of Eomes at the onset of ILC1 maturation substantially blocked cytolytic NK cell development, shifting the balance toward helper-like ILC1s (30). TGF-β has been shown to suppress Eomes in a tissue-specific population of ILC1s within the salivary glands, with genetic abrogation of TGF-βRII in NKp46^+^ cells significantly reducing ILC1 numbers, expression of CD49a, CD103, CD69a, TRAIL, and CD73 (31). Similarly, NK cells can convert into cells that resemble ILC1s due to the presence of TGF-β in a tumor microenvironment (32, 33). These ILC1s were devoid of cytotoxic activity and expressed Hobit and TIGIT, transcription factors known to suppress NK cell function.

In humans, CD127^+^ ILC1 have been shown to differentiate into ILC3-like cells in the presence of CD103^+^ dendritic cells secreting IL-2, IL-23, and IL-1β, which was enhanced in the presence of retinoic acid and dependent on the transcription factor RORγt; such reprogramming was fully reversible in the presence of IL-12 and IL-18 (34). Studies into the plasticity of ILC1s have been confounded since an extensive mass cytometry study of healthy and inflamed tissues revealed that whereas human ILC2 and ILC3 populations could be phenotypically delineated into separate clusters, ILC1s were identified throughout other cell populations (35, 36). This was compounded by the fact that ILC1 inherently lack a specific lineage marker (35, 36). ILC1s are particularly prominent during inflammatory conditions but whether they are true ILC1 or arise predominantly from ILC3 under the influence of IL-12 or ILC2 exposed to IL-1β still remains to be fully resolved.

## ILC2 Plasticity

ILC2s are in general defined as lineage negative cells, which are positive for Thy1, Sca-1, GATA-3, ST2 (IL-33 receptor), ICOS, CD44, CD25, CD127, KLRG1, and c-kit low to positive (37). ILC2 cells display great heterogeneity, with single-cell sequencing of intestinal ILC2s at steady state identifying several subgroups displaying differential gene expression patterns that differed to lung ILC2s (38, 39). Whereas, natural ILC2s produce a type-2 cytokine profile, plastic inflammatory ILC2s can coproduce both type 2 and the ILC3-characteristic cytokine IL-17. Interestingly, inflammatory KLRG1^hi^ ILC2s, which migrated to the lungs upon IL-25 challenge or following infection with the hookworm *Nippostrongylus brasiliensis* or the fungus *Candida albicans*, expressed both GATA3 and RORγt and could produce IL-17 as well as IL-13 *in vitro* (10). Notch signaling has been shown to induce *RORC* expression and drive IL-13/IL-17 co-producing ILC2 cells during house dust mite induced airway inflammation in mice (40). ILC2s from healthy human donors also express low amounts of RORγt and can co-produce IL-13 and IL-22 demonstrating that key functions of ILC2 and ILC3 subsets can co-exist in one cell but appear to be exquisitely balanced by the inflammatory milieu.

Human ILC2s activated by IL-1β have been shown to convert into IFN-γ producing ILC1s by induction of low levels of T-bet and IL-12RβII expression (41, 42). IL-12 stimulation appears to act as a rheostat in directing the ILC1 or ILC2 response, although IL-12 alone is not enough to induce this functional plasticity, a process that can be reversed by exposure to IL-4 (41, 42). In patients with severe COPD, there was elevated IL-12 and an accumulation of IFN-γ^+^ ILC2s (43). ILC2 were also shown to upregulate T-bet expression and acquire an ILC1 phenotype in intestinal samples from Crohn's disease patients (44). Even in healthy human donors, a small subset of ILC2 cells have the capacity to co-produce IL-13, IFN-γ, and IL-22 (45, 46).

## ILC3 and LTi Plasticity

Previously, LTi cells were categorized as a subset of ILC3s, although more recent studies have resolved that they are separate populations. Plasticity between the two populations, with the identification of LTi-like ILC3s and the lack of NCR expression on LTi cells that is confounded by its heterogeneous expression on ILC3s, supports the collective discussion of their plasticity. RORγt^+^ ILC3 cells have been shown to co-express T-bet, produce IFN-γ and differentiate into ILC1 cells in response to inflammation. Purified NKp44^+^ ILC3s from the murine fetal intestine when cultured with IL-2, IL-23, and IL-1β differentiate into ILC3, however when exposed to IL-2 and IL-12 they acquire the ILC1 phenotype, losing expression of NKp44 and c-kit (47). The switch also appears bi-directional, as IL-2 and IL-23 stimulation of these ILC1 cells, although maintaining *RORC* expression, caused a significant reduction in the Th1-specific transcription factor T-bet encoded by *TBX21* (47). Human natural killer 22 (NK-22) cells that express IL-22, now defined as ILC3 cells by current nomenclature, have demonstrated similar loss of IL-22 production and acquisition of IFN-γ expression (48–50). Culturing of tonsillar NK-22 cells, in the presence of IL-2 considerably modified the NK-22 cell cytokine profiles, with IL-2 promoting IFN-γ secretion and reducing secretion of IL-17 and IL-22 (49). One mechanism driving ILC3 plasticity derives from the levels and availability of the transcription factor T-bet, a critical mediator in lineage commitment of CCR6^−^ RORγt^+^ ILCs. A distinct subset of IL-22 producing ILC3s, which also express NKp46, reside in the gut and develop through T-bet regulation (12, 51). Mice exhibiting loss of T-bet expression through genetic ablation developed CCR6^−^ RORγt^+^ ILC3s but failed to develop NKp46-expressing RORγt^+^ ILCs (NK-22 cells) and could not produce IFN-γ (51).

Environmental cues from commensal microbiota have been shown to be critical in upregulating T-bet expression. Indeed, specific pathogen free (SPF) mice were shown to have greater numbers of T-bet^+^ NKp46^−^ RORγt^+^ ILC numbers compared to germ-free mice, with a corresponding decrease in NKp46^−^CCR6^−^T-bet^−^RORγt^+^ ILCs (51). Although studies in the lung during either homeostatic or inflammatory conditions remain to be investigated, gut microbiota has been shown to stabilize RORγt expression in LTi cells in mice treated with antibiotics, with IL-12 and IL-15 identified as the main drivers of RORγt loss (52). Similar to the lung, in the intestine, the process of transdifferentiation appears to be a requirement of ILC function during inflammation. Indeed the production of IFN-γ by T-bet-expressing CCR6^−^ RORγt^+^ ILCs was shown to be essential for the release of mucus-forming glycoproteins to protect the epithelial barrier during *Salmonella enterica* infection in the intestine and in Crohn's disease patients which display higher frequencies of ILC1s (34, 51). Although loss of RORγt from LTi cells and IFN-γ release leads to potent induction of colitis (52). These results reveal how intestinal microbiota can impact upon transcription factor gradients and act as rheostats for ILC functional programming, identifying how critical the tissue microenvironment is for shaping the immune response. These studies raise the exciting possibility that the lung microbiota may similarly affect ILC transdifferentiation in lung homeostasis and inflammation, which now needs investigation in the chronic disease setting, especially to further our understanding of the pathology underlying exacerbations.

## ILCs in Chronic Lung Disease

ILCs only represent a small fraction of the total immune cells in the lung, however it is clear that they play key roles in protection of the pulmonary system against a diverse array of microbes. While critical for respiratory immunity, there is now increasing evidence that these cells are implicated in chronic lung diseases and may represent disease biomarkers or targets for therapeutic intervention. The following sections will describe what is currently known about the role of these cells in chronic lung diseases.

### Asthma

Asthma is a chronic disease of the airways characterized by airway hyper-reactivity, bronchoconstriction and mucus over-production, and is classically associated with type 2 inflammation with Th2 cells thought to be the predominant source of these cytokines. Asthma is a highly heterogeneous disease with numerous endotypes including allergic and non-allergic disease (53) and it can be stimulated by many different triggers including airborne allergens and irritants, respiratory infection, cold air, and exercise. It is now appreciated that the disorder involves more than the adaptive arm of the immune system, with ILC2s, which are known to produce type 2 cytokines, being highly implicated as key players in asthmatic lung inflammation. Several recent reviews have highlighted the roles of ILCs in allergic asthma (13, 54); thus we will only touch on this briefly in this review, and instead focus on the contribution of ILCs in human studies of chronic asthma.

Several studies have identified a critical role for ILC2s in allergic asthma. ILC2 express IL17RB (IL25R) and ST2 (IL33R) and respond to intranasal administration of IL-25 and IL-33 producing IL-13 and IL-5, which contribute to the development of airway hyper-reactivity, eosinophilia, and airway inflammation (55–58). Both IL-25 and IL-33 are involved in type 2 immunity and are produced by airway epithelial cells. Allergen challenge greatly elevated IL-33 production, eliciting IL-13-expressing ILC2s in the lung and airways to induce airway hyper-reactivity (59). IL-25 or IL-33 challenge can induce the activation and accumulation of ILC2s within the lung-draining mediastinal lymph nodes, although in lung tissue, IL-33 administration induced a more sustained response. Indeed, only IL-33 could exert a direct chemotactic effect on ILC2s, which was mediated through the activation of ERK1/2, p38, Akt, JNK, and NF-κB (60), although IL-25 also stimulates several other cell types including Th2 cells and iNKT cells (61) which together would further amplify lung inflammation. In studies investigating the steroid responsiveness of ILC2s in allergen-challenged mice, reduced numbers, and increased cell death following dexamethasone treatment was observed suggesting that they are sensitive to glucocorticosteroids in eosinophilic asthma (62).

Most studies on the role of ILCs in asthma utilize experimental models that are challenged by short-term exposure to allergens, therefore the role of ILC2s in chronic asthma remains largely unknown. Although, in line with the discovery of a pathogenic role of ILC2s in experimental asthma, ILC2s are also elevated in number and activation status in patients with chronic asthma. ILC2s accumulate in the lung tissue, airway mucosa and the sputum of asthmatic patients and the numbers are further increased when challenged with allergen (63, 64). Indeed, PBMCs isolated and exposed to IL-25 and IL-33 produced a greater amount of IL-5 and IL-13 than healthy controls (65). Increased numbers of ILC2s in asthmatics correlate with eosinophilia and asthma severity, suggesting a link to disease pathogenesis (65, 66). A transcriptomic study recently revealed a link between expression profiles of ILC2s and allergic asthma susceptibility genes in mice and humans including RORA, SMAD3, GATA3, IL13, IL18R1, and IL1RL1, suggesting a role for ILC2 in regulating susceptibility gene expression (67).

The role for ILC3s still requires further investigation, but IL-17^+^ILC3s were found to be elevated within the BAL of asthma patients compared to healthy subjects (68). In line with this finding, genetic profiling of patients with adult-onset asthma, which is often more severe and associated with a poorer prognosis than childhood-onset asthma, revealed that ILC3 gene signatures, along with pathways involving eosinophilia and mast cells, were highly enriched in nasal brushings, sputum, and endobronchial brushings (69). IL-22 production is elevated in allergen-challenged mice and associated with airway hyper-responsiveness (70), and increased IL-22 expression has also been detected in the serum of asthma patients (71). A single study has identified Lin^−^CD90^+^Sca-1^+^ILCs to be the producers of IL-22 but further studies are required to determine if ILC3s also contribute to its production (72). Overall, ILC2s are likely to play a key role in the initiation and propagation of type 2 responses in the lung which may involve crosstalk with conventional T cells. Further evidence has demonstrated crosstalk between commensal bacteria, intestinal mucosal dendritic cells and IL-22-producing ILC3s in establishing the pulmonary immune system of newborn mice and promoting their resistance to pulmonary infections and suggesting that they may play a protective role, preventing the development of lung disorders such as asthma (73). It seems likely that a more intricate cellular network involving IL-17 and IL-22 producing ILC3s and γδ-T cells as well as NKT cells may also exist in the asthmatic lung, as these cells are known to induce airway hyperresponsiveness in the absence of adaptive immunity.

### Chronic Rhinosinusitis

Chronic rhinosinusitis is a persistent inflammatory disease of the nasal passages and sinuses that arises through an abnormal host response to environmental stimuli at the nasal and sinus mucosa and in its most severe form, is associated with the development of nasal polyps. Since cytokines such as IL-25, IL-33, and TSLP and their receptors are involved in the disease (74), by inference, this implicates ILCs in its pathogenesis. While studies in this area are limited, an increased proportion of ST2^+^ ILCs have been observed in the sinonasal mucosa from patients presenting with chronic rhinosinusitis and nasal polyps (75). ST2^+^ ILCs, now identified as ILC2s (76), have been shown to be increased in number and correlate with worsening nasal symptoms (77). A recent study found that ILC2s are not only increased in number, but they also exhibit an activated phenotype in chronic rhinosinusitis patients (78), suggesting that ILCs play a significant role in disease manifestation.

### COPD

COPD is a chronic inflammatory lung disease that is characterized by airflow limitation and triggered by an exaggerated inflammatory response to noxious stimuli such as cigarette smoke. ILCs can drive disease in the lung through accumulation and/or an alteration in their subset composition. In COPD patients, IL-12 signatures and the accumulation of ILC1s are elevated. IL-12 induces the conversion of ILC2s into IFN-γ-producing ILC1s thus contributing to the type 1 inflammation associated with COPD (43). The increase in ratio of ILC1:ILC2 has also been shown to correlate with lung function decline and increased disease severity (79). Gene expression profiling studies on a small subset of samples from patients with centrilobular emphysema showed that genes expressed by NK, LTi, and ILC1 cells were enriched in the inflammatory cell infiltrate, suggesting that emphysematous destruction is driven by a Th1-type response (80).

Recent studies on experimental mouse models have provided the first evidence that ILC2s may participate in COPD pathogenesis. Following cigarette smoke exposure, ILC2-deficient mice (*Ror*a^*fl*/*fl*^*Il7r*^Cre^) developed similar levels of airway inflammation to wild type mice although the loss of ILC2s appeared to protect from cigarette smoke-induced emphysema (81). However, ILC2-deficient mice had increased IL-33 and IL-13 expression and substantial collagen deposition identifying a role for ILC2s in airway fibrosis and lung remodeling processes (81).

The release of the major stimulators of ILC2s, TSLP, IL-25, and IL-33, as a result of epithelial cell injury in both asthma and COPD, provide evidence of ILC2s in airway remodeling. IL-33 expression increases in basal epithelial progenitor cells in patients with COPD, and has been linked to increased IL-13 and mucin gene 5AC expression (17), suggesting a role for ILC2s in the airway disease components of COPD such as mucus hypersecretion, airway hyper-responsiveness and fibrosis.

When considering cell-intrinsic mechanisms regulating ILC2 function, the enzyme arginase-1 (Arg1), which is considered a classic alternatively activated macrophage marker, was recently identified as a critical mediator in the control of a metabolic program within the ILC2 subset in the lung and the development of type 2 inflammation. Arg1 is known to promote collagen synthesis and fibrosis to support wound healing in the lung. In lung tissue from patients with COPD and idiopathic pulmonary fibrosis (IPF), Arg1 was found to be elevated in ILC2s although levels between these two groups did not differ significantly, suggesting Arg1 expression is a general inflammatory signature of these cells (82). Loss of ILC-intrinsic Arg1 activity prevented a robust ILC2 response and reduced the emphysematous phenotype of COPD, identifying a role for Arg1 and its control of ILC2 responses in chronic lung inflammation (82).

A role for ILC3s in COPD has been implicated as IL-17 is a key mediator of neutrophilia in COPD. Indeed, COPD patients have a greater number of IL-17- and IL-22-expressing cells in bronchial biopsies (83) and a recent study identified the presence of both natural cytotoxicity receptor (NCR)^+^ and NCR^−^ ILC3 subsets in the lungs of COPD patients (14). However, a higher frequency of NCR^−^ ILC3s were found compared to healthy controls, as well as elevated IL-17 and IL-22 production by ILC3s. Furthermore, the recent identification of NRP1^+^ as a marker of LTi-like ILC3 cells in the lungs of smokers and COPD patients, suggests these cells may play a key role in the formation of ectopic pulmonary lymphoid aggregates and the promotion of airway angiogenesis (84).

The lung tissue destruction that occurs in COPD highly implicates cytotoxic lymphocytes such as NK cells in this damaging process. While early studies in COPD patients, suggested that NK cells in the circulation were reduced in number and had compromised phagocytic activity (85, 86), further studies showed that there was an increase in the proportion and cytotoxic activity of NK cells in the BAL or induced sputum from smokers with COPD compared to healthy smokers (86, 87). Moreover, NK cells isolated from the lung tissue of patients with severe COPD had increased cytotoxicity which correlated with decreased pulmonary function (88). Mouse studies confirmed these findings, with cigarette smoke exposure increasing the number of NK cells within the lung, which also displayed an activated phenotype (89–91). The upregulation of epithelial-derived IL-33 by cigarette smoke exposure during a viral exacerbation was shown to increase both NK cell recruitment and effector cytokine responses through the upregulation of ST2, although interestingly, ILC2 cells conversely downregulated ST2 expression (92). Ligands for NKG2D activatory receptors on NK cells have been found to be induced on stressed lung epithelium, which may provide a mechanism to promote NK cell activation in lung disease (93), and in support of this, sustained NKG2D activation in a transgenic mouse model was sufficient to cause pulmonary emphysema (94). This finding was reinforced by the demonstration that cigarette smoke induced the sustained expression of NKG2D ligands on mouse epithelium and strengthened by the same observation in patients with COPD (94).

### Exacerbations of COPD and Asthma

Asthma and COPD are the most common chronic airway diseases, and exacerbations, which are episodes of acute worsenings of symptoms and airflow obstruction, contribute greatly to disease morbidity and mortality. The major cause of disease exacerbations are viral infections of the respiratory tract, particularly those involving rhinovirus. In respiratory viral infection, ILCs accumulate in lung tissue and play important roles in host defense, tissue integrity and the maintenance of homeostasis in the lung (5). However, they are also implicated in asthma and COPD exacerbations following lung infection (43, 95). It has been well-characterized that ILC2-activating cytokines, IL-25, and IL-33, are released by airway epithelial cells upon viral infection, where they play key roles in disease exacerbations through enhancement of type 2 inflammation. These cytokines induce ILC2 accumulation in the lung, which in turn release IL-5 and IL-13, leading to eosinophil recruitment, mucus production and macrophage polarization (96). BAL eosinophil numbers and IL-5 and IL-13 mRNA expression in lung tissue were greatly reduced when ILCs were depleted by anti-CD90.2 antibody (97). Thus, intervening in the action of ILC2s and their associated type 2 cytokines during a viral infection may be an effective strategy to manage viral exacerbations.

Cigarette smoking, which is one of the major risk factors for COPD, is known to alter the lung immune response to infection (98). Smoke-exposed mice, which show an upregulation of IL-33 expression in the lung epithelium, are completely protected from influenza virus-induced exacerbation when deficient in either IL-33 or its receptor ST2 (92). Prior exposure to smoke was shown to compromise the ability of the lung to mount a Th2 response and subsequent production of IL-13 from ILC2 cells, which led to an exacerbated Th1 proinflammatory response. In support, a study of pulmonary biopsies from patients revealed that elevated IL-33 in lung tissue sections correlated with severity of COPD (92).

Trans-differentiation of ILCs is also linked to lung disease exacerbations. In response to pathogens that trigger COPD exacerbations (influenza A virus, respiratory syncytial virus, *Staphylococcus aureus*, non-typeable *Haemophilus influenzae*), mouse ILC2s substantially lowered their expression of the transcription factor GATA-3, exhibited an increase in expression of T-bet and switched to interferon-γ-producing ILC1s (36, 43).

In mice, IL-12 and IL-18 were involved in the trans-differentiation program which occurred during infections where ILC2s were observed to accumulate and acquire ILC1 effector functions (99). Extending these studies to human samples, IL-12 could also induce the plasticity of ILC2 sorted from peripheral blood, reprogramming them into ILC1 cells (41). Interestingly, the frequency of ILC1 cells in patients with COPD was linked with disease severity and exacerbation susceptibility (43). These studies highlight the unfavorable effect of ILC2 plasticity on anti-viral immunity and its adverse effect on COPD during an exacerbation. In this context, it is critical we now learn how to control the plasticity of lung ILC2s, which may help to manage exacerbations and slow the progression of chronic lung disease.

### Pulmonary Fibrosis

Pulmonary fibrosis is a feature of lung diseases such as asthma, COPD, cystic fibrosis, and idiopathic pulmonary fibrosis (IPF). IPF is a progressive, fibrotic disease of the lung with an unknown etiology and an extremely poor prognosis. It is characterized by a dysregulated wound healing response, the over-production of profibrotic factors such as IL-13 and TGF-β and the activation of myofibroblasts that leads to extracellular matrix accumulation. While this is an understudied area, type 2 responses have been implicated in the pulmonary fibrosis phenotype (100) and therefore by association, ILC2 cells may play a significant role. Levels of the cytokines IL-33 and TSLP, which are inducers of ILC2s, have been found to be significantly elevated in the BAL of patients with IPF relative to normal control subjects (18). IL-25 has also been shown to be elevated in the BAL fluid of IPF patients with a corresponding increase in numbers of ILC2 cells compared to healthy subjects (101).

The role of ILC2s in pulmonary fibrosis and airway inflammation is supported in animal models where the production of IL-13 by IL-25-elicited ILC2s was sufficient to drive collagen deposition in the lungs of bleomycin-challenged mice (101). Likewise, intranasal delivery of recombinant IL-25 to mice caused airway inflammation, connective tissue growth factor (CTGF) and TGF-β1 production and subsequent pulmonary fibrosis (102). Similar observations have been made for IL-33, with elevated levels of IL-33 detected in lung tissues during fibrosis or intestinal epithelium of patients with pulmonary fibrosis or fibrotic colitis, respectively, and in the liver of mice with hepatic fibrosis (103). Mechanistically, it has been proposed that IL-33 drives fibrosis by inducing the production of the profibrotic cytokine IL-13 in ILC2s, macrophages, and eosinophils.

NK cell activation may protect against lung fibrosis through the production of IFN-γ. In murine models of bleomycin-induced pulmonary fibrosis, CXCR3 deficiency resulted in loss of NK cell recruitment to the lung and subsequent IFN-γ production resulting in increased pulmonary fibrosis (104). The expression of NKG2D, an activatory receptor on NK cells, was shown to be reduced on NK, NKT, and γδ-T cells isolated from the BAL of patients with IPF, suggesting NK function may be impaired in pulmonary fibrosis (105, 106). Interestingly, the expression of the NKG2D ligand, MICA, is upregulated on fibroblasts and the epithelial cells within the lung of IPF patients suggesting ligand concentrations, in addition to cytokine milieu, may play a role in directing NK cell function and the concerted modulation of NKT and γδ-T cells during progression of chronic lung disease (106).

**Figure 2:**
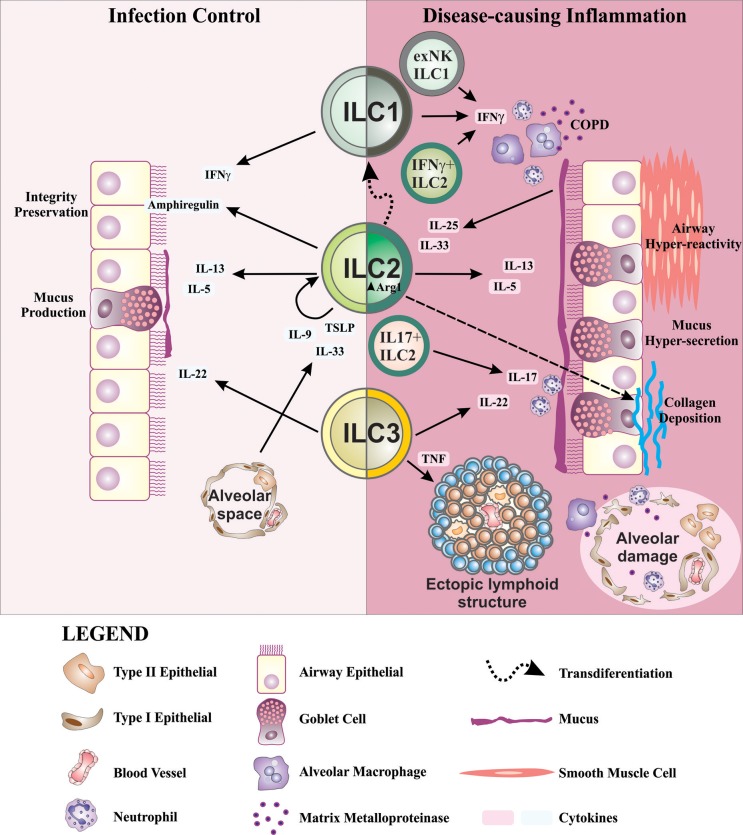
Schematic representation of ILCs in lung health and disease. ILC2s are important for the preservation of airway barrier integrity during homeostasis. During inflammation in response to allergens, IL-33, and TSLP produced by alveolar type II epithelial cells are known to drive IL-9 production in ILC2s. Autocrine IL-9 promotes IL-5 and IL-13 production which drives mucus production from goblet cells in the airways. ILC2s also produce amphiregulin during infection to activate epithelial repair responses. IL-22, an important factor for the maintenance of epithelial barrier function, mucus production and tissue repair, is predominantly produced by ILC3s. In disease settings, IL-25 and IL-33 from airway epithelial cells induce IL-13 and IL-5 production from ILC2s which contribute to airway hyper-reactivity, mucus overproduction, and airway inflammation in asthma. Further dysregulation of ILC2s through increased expression of Arg1 promotes collagen synthesis and deposition causing lung fibrosis. In addition to the contribution of IFN-γ^+^ from ILC1s in recruiting and activating alveolar macrophages in COPD, there is accumulation of IFN-γ^+^ ILC2s. Similar alterations in ILC phenotype are seen in asthma with the production of IL-17, primarily produced by ILC3s, co-expressed with IL-13 by ILC2s causing neutrophilia in COPD and asthma. Airway remodeling, an important feature in asthma and COPD, may in part be supported by LTi-like ILC3s and their participation in the formation of ectopic lymphoid structures. The above findings demonstrate a highly complex interplay of ILCs, cytokines and other inflammatory mediators within the lung. The highly plastic nature of ILCs, which follow cues from the inflammatory milieu, causes them to become dysregulated during chronic lung disease due to an overt and persistent onslaught of inflammatory mediators.

ILCs, in addition to participating in lung homeostasis, have now been shown to also contribute to a number of lung pathologies. ILCs become dysregulated in chronic lung disorders including asthma, COPD, chronic rhinosinusitis, and pulmonary fibrosis, which in part may be due to the highly heterogeneous nature of ILCs, and their ability to respond to changing local tissue environmental conditions by altering their traits and functional attributes (Figure 2). Further studies are now required to fully appreciate how ILCs contribute to the immunopathology of chronic lung disease.

## Role of Unconventional T Cells in Chronic Lung Disorders

In addition to ILCs, other innate-like unconventional αβ- and γδ-T cells have recently emerged as central players in pulmonary immunity. Both MR1-restricted MAIT cells and CD1d-restricted NKT cells express a surface receptor comprised of a semi-variant TCR-α chain complexed with a TCR-β chain of limited repertoire. MAIT cells share some similarities with NKT cells including restriction by non-classical MHC molecules and expression the transcription factor PLZF. Unconventional γδ-T cells differ from conventional T cells due to the expression of a γδ TCR of limited TCR diversity. Unlike conventional T cells that recognize antigens complexed with MHC class I and II molecules, unconventional γδ-T cells recognize lipids, metabolites, and modified peptides presented by MHC class Ib and MHC class-I-like molecules (107). The innate-like phenotype of unconventional T cells is exemplified by their ability to secret cytokines and chemokines without prior antigen exposure upon thymic egress. Similarly, γδ-T cells, which mature and exit the thymus prior to RAG recombination of the γδ TCR, are largely effector-like cells and express a semi-variant TCR. Whereas, MAIT and NKT cells each comprise ~2% of the T cell population in the lung, γδ-T cells are estimated to represent up to 8–20% of all resident pulmonary lymphocytes (107, 108). Although there is a growing understanding of the role of MAIT, NKT, and γδ-T cells in host protection from pathogens, at present their role and relevance in lung disease is largely unknown.

Unconventional T cells respond to microbial pathogens within the lung. NKT cells become activated in response to microbial CD1d-restricted lipids and upon exposure to inflammatory cytokines such as IL-12 (109, 110). Similarly to NKT cells, which respond predominantly to bacterial challenges, MAIT cells are activated by bacteria and yeasts and produce TNF-α and IFN-γ to control infection (111). γδ-T cells sense cellular stress-induced signals through TCR-dependent and -independent pathways. Activation through the γδ-TCR can occur through non-classical MHC molecules including T10/T22 and CD1 family members as well as butyrophilin 3A1 and viral glycoproteins (112). γδ-T cell ligands are still largely undefined but γδ-T cells have been shown to respond to phospholipids, viral proteins, and stress-induced molecules. γδ-T cells also can respond to pathogen-associated molecular patterns through expression of numerous pattern recognition receptors on their surface as well as NK receptor ligands Rae1 and MICA/B (113). Lung inflammation drives γδ-T cells to secrete IFN-γ and TNF-α and they have also been shown to be a potent source of IL-17. There is an obvious overlap in positioning, ligand recognition, activation, and effector function between the unconventional T cells. Unconventional T cells all express cell surface receptors for the detection of microbial products, which unlike the ILC population within the lung, gives them specificity to directly respond to infectious agents entering the lung rather than to a diverse inflammatory milieu. Given their cytolytic capacity and cytokine profiles, unconventional T cells appear to be a specialized innate-like group of cells that are poised to act in early host defense to a range of pathogens and their bi-products. It also suggests that unconventional T cells may play a specific and perhaps intersecting role during infection of the chronically inflamed lung, contributing to exacerbations.

### NKT Cells

The most widely studied subset of NKT cells are invariant NKT (iNKT) cells, expressing a TCR with limited diversity that responds to microbial glycolipid antigens. Upon activation, iNKT cells rapidly produce IFN-γ and IL-4 among other cytokines, indicating that they act early in the immune response and can coordinate both the innate and adaptive arms.

The role of iNKT cells in asthma so far is inconclusive [reviewed in (114)]. Bronchial asthma is classically thought to be a Th2 cell-associated disease characterized by CD4^+^ Th2 cells producing IL-4, −5, and −13. Yet, there are reports that a significant proportion of lymphocytes in the blood are CD1d-restricted CD4^+^ iNKT cells that inversely correlate with atopic indexes in asthmatic patients (115, 116). However, the inverse has also been reported in the BAL where fewer than 2% of T cells were CD4^+^ iNKT in patients with asthma, a number which did not differ significantly from the number found in healthy subjects (117). Nevertheless, using NKT cell deficient mice it has been shown that the absence V_α_14*i* NKT cells protects mice from developing allergen-induced airway hyper-reactivity (118). Thus, much more work is required to fully appreciate the contribution of iNKT cells to asthmatic airway disease.

In COPD, the role of iNKT cells is as confounding with CD4^+^ iNKT numbers in the BAL and sputum unchanged, although a conflicting study reported that the frequency of iNKT cells in the peripheral blood was significantly reduced in subjects with COPD compared to healthy individuals and further reduced in those COPD patients with exacerbations (117, 119). The converse trends between the blood and BAL were also seen with respect to CD4^+^ cells in a patient study of smokers with COPD. Smoking was found to increase the number of CD8^+^ T cells and CD8^+^ NKT-like cells in the BAL of COPD patients with a concurrent reduction of CD4^+^ cells in the BAL and increase of CD4^+^ in the blood as previously reported (120, 121), while another study found an increase in iNKT and NKT-like cells in BAL fluid from current smokers but not COPD patients (121). Indeed, the role of iNKT cells in COPD may more relate to exacerbations, which can be caused by infection with respiratory viruses and other pathogens. iNKT cells have been shown to contribute to COPD following viral challenge with Sendai virus and subsequent glycolipid presentation by dendritic cells, which leads to the secretion of IL-13 and alternative activation of alveolar macrophages (122). This identifies a possible role for iNKT cells in viral exacerbations of COPD, which may extend to other chronic lung diseases.

iNKT cells have been shown to induce airway hyper-responsiveness in the absence of adaptive immunity, which would suggest that iNKT cells and ILCs may interact within the lung. A study has addressed this using glycolipid antigens in a murine model of asthma. iNKT cells activated by glycolipid antigens stimulated the production of IL-33 from alveolar macrophages which in turn activated ILC2 to produce IL-13 and contribute to airway hyper-responsiveness (123). NKT cells themselves have been shown to be a source of IL-33 during influenza infection (19). Taken together, these results suggest that iNKT crosstalk in the lung is highly complex and involves other innate cells such as ILC2s.

### MAIT Cells

In humans, MAIT cells are in abundance in the peripheral blood and comprise 10% of lung mucosal T cells, which in addition to the limited diversity of the MAIT-TCR means that early within the immune response, MAIT cell responses significantly outnumber those from conventional αβ-T cells. MAIT cells display an effector-memory phenotype without prior clonal expansion and can secrete a range of pro-inflammatory cytokines including TNF-α, IL-17, and IFN-γ and also IL-4 upon TCR ligation (124–126).

Circulating MAIT cell numbers have been found to be significantly reduced in patients with COPD, correlating with disease severity and inflammatory activity (127, 128). Exacerbations are major drivers of morbidity and mortality in COPD. Since exacerbations can be driven by bacterial infection, and MAIT cell activation is elicited by precursors and derivatives of the riboflavin biosynthetic pathway conserved in bacteria and yeast, there is potential for their involvement in COPD exacerbations. Macrophages stimulated with live non-typeable *Haemophilus influenzae* (NTHi), the most common airway-colonizing bacterium in COPD, promoted a potent IFN-γ response from MAIT cells, providing strong evidence that NTHi is a target of MAIT cell immunity. Interestingly, MAIT cell number and immune responses were significantly impaired by corticosteroid treatment, suggesting that failure of MAIT cell immunity may mediate COPD immunopathology during infection (127). MAIT cell frequencies were markedly reduced in the blood and lung tissues of severe asthma patients, which was also related to corticosteroid treatment (129).

MAIT cells as well as NK cells and ILCs have been shown to be involved in asthma. MAIT cells, activated NK cells (CD69^+^), ILC1, ILC2, and ILC3 cells have all been shown to positively correlate with each other and with reduced airflow in asthmatic patients (130). This would suggest an underlying bacterial challenge could be involved in the increase in MAIT cell numbers, although an increase in all ILC populations and their mixed response seems contradictory. In response to viral challenge by influenza A virus and the bacteria *Staphylococcus aureus* and NTHi, all of which are COPD-associated triggers, ILC populations were altered. Each pathogen induced ILC reprogramming in the lung by inducing the loss of GATA-3 expression, while increasing IL-12RβII, IL-18Rα, and T-bet expression (43). Similarly, exposure to cigarette smoke induced a loss of GATA-3 expression and emergence of an ILC1 population. Interestingly, exposure to cigarette smoke combined with viral infection augmented the ILC phenotypic switch, suggesting the response was due to pathogenic or environmental insults (43). Understanding how MAIT cells contribute to these exacerbations and support ILC plasticity may define their role in pulmonary immunity and the immunopathology of chronic respiratory diseases.

### γδ-T Cells

γδ-T cells are tissue-resident cells primarily located at mucosal sites within the body; in the lung they have been characterized as CD4 and CD8 double negative cells selectively expressing Vγ6, Vγ1, and Vγ4 gene segments (131). The γδ-T cells residing in the lung are potent producers of IL-17 whereas γδ-T cells expressing different Vγ gene segments in the skin, gut, liver, spleen, uterus, and peripheral blood can produce IL-17 or IFN-γ (131). The abundance of γδ-T cells in the lung supports tissue homeostasis, although γδ-T cells have also been shown to play critical roles in bacterial clearance and the prevention of inflammation and lung fibrosis (132).

Little is known about the roles of γδ-T cells in chronic lung diseases such as COPD except that they are an important component of tissue injury and remodeling. The specific localization of γδ-T cells in epithelial and mucosal tissues and their role in protecting and maintaining airway function, suggests that they are likely participants in chronic airway diseases. Cigarette smoke exposure is the major cause of COPD and has been directly implicated in neutrophil variant asthma, asthma-COPD overlap syndrome, interstitial lung disease, rheumatic lung disease, and infectious exacerbations of these conditions (87, 133–135). Studies have shown elevated numbers of γδ-T cells in the bronchial glands, lung parenchyma, peripheral blood and BAL of smokers compared to never smokers (136–138). Early reports demonstrated that γδ-T cell percentages in the BAL and peripheral blood increased in COPD patients compared to healthy controls and were further amplified by smoking (139, 140). In contrast, a more recent study reported significantly lower relative and absolute numbers of γδ-T cells in the sputum and BAL of patients with COPD than those with asthma or healthy subjects, which negatively correlated with FEV1 and smoking pack years (141). This finding contradicted earlier publications but failed to stratify patient subgroups due to small sample size whereas the previous studies may have been confounded by inhaled steroid and bronchodilator therapies, and thus, much more work is required to fully appreciate the contribution of γδ-T cells in COPD.

In many cases, airway infections can cause COPD exacerbations, which are an acute worsening of respiratory symptoms. IL-17 producing cells, in particular Th17 cells, play distinct roles in host defenses against diverse pathogens (142). Pathogens that invoke an IL-17 response involve innate immune cells such as γδ-T cells, NKT cells, MAIT cells, and ILC3s, as well as adaptive Th17 cells. Persistence of some bacteria such as *Pseudomonas aeruginosa* within the lower airways is common in patients with cystic fibrosis, non-cystic fibrosis bronchiectasis, and COPD. In a murine model of chronic pulmonary infection with *Pseudomonas aeruginosa*, there was a significant expansion of IL-17^+^ cells in lung homogenates and of these, 50% were CD3^−^ IL-17^+^ ILC3s, likely to be LTi cells and 50% were CD3^+^ T cells, split equally between γδ-T cells and Th17 cells, demonstrating a diverse range of cellular sources of IL-17 in chronic respiratory infection (143). Why a spectrum of IL-17-producing cells are generated during pulmonary infection and the roles of these different cell types, remains to be determined.

**Figure 3:**
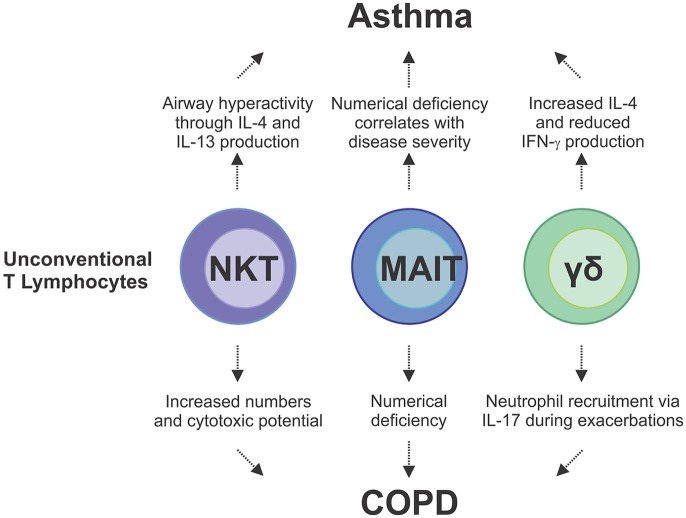
Unconventional lymphocytes are implicated as pathogenic mediators of asthma and COPD and disease exacerbations. In asthma, IL-4 and IL-13 cytokines produced by NKT cells are directly involved in disease development which is compounded by additional IL-4 production from γδ-T cells. Conversely there is a reduction in IFN-γ producing γδ-T cells and MAIT cells, which correlates with disease severity in asthma and COPD. In COPD, there is an increase in NKT cell numbers and cytotoxicity in the lungs, and IL-17-mediated neutrophilia, which is driven by γδ-T cells, both of which contribute to lung damage. These studies demonstrate the complex interplay and multifaceted contributions from the different types of unconventional T cells to chronic lung disease.

Just as cigarette smoke has been shown to increase the number of IL-17A^+^ NKT cells in the lung, alternative cellular sources of IL-17A include NK cells and γδ-T cells which have been shown to become potent producers of IL-17A upon cigarette smoke exposure, with the frequency of IL-17^+^ γδ-T cells significantly increasing in number (144). Clinical studies and experimental models of viral-induced COPD exacerbations provide strong evidence of ineffective anti-viral immunity in response to cigarette smoke. Although increased γδ-T cell numbers and production of IL-17A in response to cigarette smoke has been shown to be protective, pneumococcal challenge of mice chronically subjected to cigarette smoke led to defective production of IL-17 from γδ-T cells (145). Similarly, in the presence influenza A, mice exposed to cigarette smoke recovered poorly from an acute infection (146). During influenza infection, γδ-T cells acquire reciprocal production of IFN-γ and IL-17A, however, cigarette smoke exposure leads to repression of *IFN-*γ transcription (146). The contribution of unconventional T cells to chronic lung disease, including asthma and COPD, is only starting to be understood (Figure 3). As most chronic lung disease patients experience exacerbations during the course of their disease, an improved understanding of how γδ-T cells as well as MAIT and NKT cells contribute to the cytokine milieu during these insults to drive ILC plasticity and alter function is now required. This may offer important new insights into the way in which innate and innate-like cells contribute to COPD and asthma exacerbations, as well as other chronic pulmonary diseases.

## Concluding Remarks

The development of innate and innate-like lymphocytes with overlapping phenotypes and functions has most likely evolved to provide robustness to the pulmonary immune system (147). ILCs and unconventional T cells may differ in cellular biology but they share common roles in tissue integrity preservation, lung homeostasis and immunity against infections. Compared with their conventional counterparts, it seems likely that ILCs and unconventional T cells have developed as specialized tissue-resident sensors to rapidly detect deviations in tissue integrity that arise from infection or injury. While it is clear that ILCs contribute to the maintenance of lung homeostasis, there is growing evidence to support the contribution of ILCs to a number of lung pathologies. ILCs have now been shown to become dysregulated in chronic lung disorders including asthma, COPD, chronic rhinosinusitis, and pulmonary fibrosis, and similarly, unconventional T cells are involved in the immunopathology of these diseases, particularly in the context of disease exacerbations. Recent advances have identified the highly heterogeneous and flexible nature of ILCs, which enable them to readily adapt to changing local tissue environmental conditions by altering their traits and functional attributes. This now introduces the question of how ILCs functionally integrate into the complex network of immune cells and stroma within the lung. In particular, the interplay and functional overlap between these innate cells and the other tissue-resident unconventional T cells requires further investigation. Whether or not these cell subsets co-regulate one another or function independently remains to be answered. We have discussed the dysregulation of ILC function during chronic lung disease, a feature that becomes more pronounced during exacerbations due to the sensitivity of ILCs to the changing lung tissue microenvironment but also in part owing to the complex interplay between ILCs and unconventional T cells within the lung. Further studies should now be directed at understanding these complex relationships, particularly in the setting of chronic lung disease, which may reveal potential treatment targets and lead to the formulation of interventions against these prevalent and debilitating diseases.

## Author Contributions

JB conceived and wrote the manuscript. MH and ML contributed to the writing of the manuscript and prepared the figures.

### Conflict of Interest Statement

The authors declare that the research was conducted in the absence of any commercial or financial relationships that could be construed as a potential conflict of interest.

## References

[1] Diefenbach A Colonna M Koyasu S. Development, differentiation, and diversity of innate lymphoid cells. Immunity. (2014) 41:354–65. 10.1016/j.immuni.2014.09.005 25238093 PMC4171710

[2] Vivier E Artis D Colonna M Diefenbach A Di Santo JP Eberl G. Innate lymphoid cells: 10 years on. Cell. (2018) 174:1054–66. 10.1016/j.cell.2018.07.017 30142344

[3] Yu YRA O'Koren EG Hotten DF Kan MJ Kopin D Nelson ER. A protocol for the comprehensive flow cytometric analysis of immune cells in normal and inflamed murine non-lymphoid tissues. PLoS ONE. (2016) 11:e0150606. 10.1371/journal.pone.0150606 26938654 PMC4777539

[4] Dutton EE Camelo A Sleeman M Herbst R Carlesso G Belz GT. Characterisation of innate lymphoid cell populations at different sites in mice with defective T cell immunity. Wellcome Open Res. (2017) 2:117. 10.12688/wellcomeopenres.13199.3 29588921 PMC5854988

[5] Monticelli LA Sonnenberg GF Abt MC Alenghat T Ziegler CG Doering TA. Innate lymphoid cells promote lung-tissue homeostasis after infection with influenza virus. Nat Immunol. (2011) 12:1045–54. 10.1038/ni.2131 21946417 PMC3320042

[6] Gasteiger Fan X Dikiy S Lee SY Rudensky AY. Tissue residency of innate lymphoid cells in lymphoid and nonlymphoid organs. Science. (2015) 350:981–5. 10.1126/science.aac9593 26472762 PMC4720139

[7] Moro K Kabata H Tanabe M Koga S Takeno N Mochizuki M. Interferon and IL-27 antagonize the function of group 2 innate lymphoid cells and type 2 innate immune responses. Nat Immunol. (2016) 17:76–86. 10.1038/ni.3309 26595888

[8] Haas JD Ravens S Duber S Sandrock I Oberdorfer L Kashani E. Development of interleukin-17-producing γδ T cells is restricted to a functional embryonic wave. Immunity. (2012) 37:48–59. 10.1016/j.immuni.2012.06.003 22770884

[9] McKenzie DR Kara EE Bastow CR Tyllis TS Fenix KA Gregor CE. IL-17-producing γδ T cells switch migratory patterns between resting and activated states. Nat Commun. (2017) 8:15632. 10.1038/ncomms15632 28580944 PMC5465362

[10] Huang Y Guo L Qiu J Chen X Hu-Li J Siebenlist U. IL-25-responsive, lineage-negative KLRG1(hi) cells are multipotential 'inflammatory' type 2 innate lymphoid cells. Nat Immunol. (2015) 16:161–9. 10.1038/ni.3078 25531830 PMC4297567

[11] Huang Y Mao K Chen X Sun MA Kawabe T Li W. S1P-dependent interorgan trafficking of group 2 innate lymphoid cells supports host defense. Science. (2018) 359:114–9. 10.1126/science.aam5809 29302015 PMC6956613

[12] Mohapatra Van Dyken SJ Schneider C Nussbaum JC Liang HE Locksley RM. Group 2 innate lymphoid cells utilize the IRF4-IL-9 module to coordinate epithelial cell maintenance of lung homeostasis. Mucosal Immunol. (2016) 9:275–86. 10.1038/mi.2015.59 26129648 PMC4698110

[13] Martinez-Gonzalez Steer CA Takei F. Lung ILC2s link innate and adaptive responses in allergic inflammation. Trends Immunol. (2015) 36:189–95. 10.1016/j.it.2015.01.005 25704560

[14] De Grove KC Provoost S Verhamme FM Bracke KR Joos GF Maes T. Characterization and quantification of innate lymphoid cell subsets in human lung. PLoS ONE. (2016) 11:e0145961. 10.1371/journal.pone.0145961 26727464 PMC4699688

[15] Hirose K Ito T Nakajima H. Roles of IL-22 in allergic airway inflammation in mice and humans. Int Immunol. (2018) 30:413–8. 10.1093/intimm/dxy010 29394345

[16] Prefontaine D Nadigel J Chouiali F Audusseau S Semlali A Chakir J. Increased IL-33 expression by epithelial cells in bronchial asthma. J Allergy Clin Immunol. (2010) 125:752–4. 10.1016/j.jaci.2009.12.935 20153038

[17] Byers DE Alexander-Brett J Patel AC Agapov E Dang-Vu G Jin X. Long-term IL-33-producing epithelial progenitor cells in chronic obstructive lung disease. J Clin Invest. (2013) 123:3967–82. 10.1172/JCI65570 23945235 PMC3754239

[18] Lee JU Chang HS Lee HJ Jung CA Bae DJ Song HJ. Upregulation of interleukin-33 and thymic stromal lymphopoietin levels in the lungs of idiopathic pulmonary fibrosis. BMC Pulm Med. (2017) 17:39. 10.1186/s12890-017-0380-z 28202030 PMC5312598

[19] Gorski SA Hahn YS Braciale TJ. Group 2 innate lymphoid cell production of IL-5 is regulated by NKT cells during influenza virus infection. PLoS Pathog. (2013) 9:e1003615. 10.1371/journal.ppat.1003615 24068930 PMC3777868

[20] Guo XJ Dash P Crawford JC Allen EK Zamora AE Boyd DF. Lung γδ T cells mediate protective responses during neonatal influenza infection that are associated with type 2 immunity. Immunity. (2018) 49:531–44.e6. 10.1016/j.immuni.2018.07.011 30170813 PMC6345262

[21] Hong JY Bentley JK Chung Y Lei J Steenrod JM Chen Q. Neonatal rhinovirus induces mucous metaplasia and airways hyperresponsiveness through IL-25 and type 2 innate lymphoid cells. J Allergy Clin Immunol. (2014) 134:429–39. 10.1016/j.jaci.2014.04.020 24910174 PMC4119851

[22] Han M Rajput C Hong JY Lei J Hinde JL Wu Q. The innate cytokines IL-25, IL-33, and TSLP cooperate in the induction of type 2 innate lymphoid cell expansion and mucous metaplasia in rhinovirus-infected immature mice. J Immunol. (2017) 199:1308–18. 10.4049/jimmunol.1700216 28701507 PMC5548608

[23] Stier MT Bloodworth MH Toki S Newcomb DC Goleniewska K Boyd KL. Respiratory syncytial virus infection activates IL-13-producing group 2 innate lymphoid cells through thymic stromal lymphopoietin. J Allergy Clin Immunol. (2016) 138:814–24.e11. 10.1016/j.jaci.2016.01.050 27156176 PMC5014571

[24] Moffatt MF Gut IG Demenais F Strachan DP Bouzigon E Heath S. A large-scale, consortium-based genomewide association study of asthma. N Engl J Med. (2010) 363:1211–21. 10.1056/NEJMoa0906312 20860503 PMC4260321

[25] Halim TY Krauss RH Sun AC Takei F. Lung natural helper cells are a critical source of Th2 cell-type cytokines in protease allergen-induced airway inflammation. Immunity. (2012) 36:451–63. 10.1016/j.immuni.2011.12.020 22425247

[26] Nagata Kamijuku H Taniguchi M Ziegler S Seino K. Differential role of thymic stromal lymphopoietin in the induction of airway hyperreactivity and Th2 immune response in antigen-induced asthma with respect to natural killer T cell function. Int Arch Allergy Immunol. (2007) 144:305–14. 10.1159/000106319 17652941

[27] Denney L Byrne AJ Shea TJ Buckley JS Pease JE Herledan GM. Pulmonary epithelial cell-derived cytokine TGF-β1 is a critical cofactor for enhanced innate lymphoid cell function. Immunity. (2015) 43:945–58. 10.1016/j.immuni.2015.10.012 26588780 PMC4658339

[28] McHedlidze T Kindermann M Neves AT Voehringer D Neurath MF Wirtz S. IL-27 suppresses type 2 immune responses *in vivo* via direct effects on group 2 innate lymphoid cells. Mucosal Immunol. (2016) 9:1384–94. 10.1038/mi.2016.20 26982595

[29] Murphy KM Stockinger B. Effector T cell plasticity: flexibility in the face of changing circumstances. Nat Immunol. (2010) 11:674–80. 10.1038/ni.1899 20644573 PMC3249647

[30] Pikovskaya O Chaix J Rothman NJ Collins A Chen YH Scipioni AM. Cutting Edge: eomesodermin is sufficient to direct type 1 innate lymphocyte development into the conventional NK lineage. J Immunol. (2016) 196:1449–54. 10.4049/jimmunol.1502396 26792802 PMC4744497

[31] Cortez VS Cervantes-Barragan L Robinette ML Bando JK Wang Y Geiger TL. Transforming growth factor-β signaling guides the differentiation of innate lymphoid cells in salivary glands. Immunity. (2016) 44:1127–39. 10.1016/j.immuni.2016.03.007 27156386 PMC5114145

[32] Cortez VS Ulland TK Cervantes-Barragan L Bando JK Robinette ML Wang Q. SMAD4 impedes the conversion of NK cells into ILC1-like cells by curtailing non-canonical TGF-β signaling. Nat Immunol. (2017) 18:995–1003. 10.1038/ni.3809 28759002 PMC5712491

[33] Gao YF Bald T Ng SS Young A Ngiow SF Rautela J. Tumor immunoevasion by the conversion of effector NK cells into type 1 innate lymphoid cells. Nat Immunol. (2017) 18:1004–15. 10.1038/ni.3800 28759001

[34] Bernink JH Krabbendam L Germar K de Jong E Gronke K Kofoed-Nielsen M. Interleukin-12 and−23 control plasticity of CD127(+) group 1 and group 3 innate lymphoid cells in the intestinal lamina propria. Immunity. (2015) 43:146–60. 10.1016/j.immuni.2015.06.019 26187413

[35] Simoni Y Fehlings M Kloverpris HN McGovern N Koo SL Loh CY. Human innate lymphoid cell subsets possess tissue-type based heterogeneity in phenotype and frequency. Immunity. (2017) 46:148–61. 10.1016/j.immuni.2016.11.005 27986455 PMC7612935

[36] Spits H Bernink JH Lanier L. NK cells and type 1 innate lymphoid cells: partners in host defense. Nat Immunol. (2016) 17:758–64. 10.1038/ni.3482 27328005

[37] Mindt BC Fritz JH Duerr CU. Group 2 innate lymphoid cells in pulmonary immunity and tissue homeostasis. Front Immunol. (2018) 9:840. 10.3389/fimmu.2018.00840 29760695 PMC5937028

[38] Nussbaum JC Van Dyken SJ von Moltke J Cheng LE Mohapatra A Molofsky AB. Type 2 innate lymphoid cells control eosinophil homeostasis. Nature. (2013) 502:245–8. 10.1038/nature12526 24037376 PMC3795960

[39] Gury-BenAri M Thaiss CA Serafini N Winter DR Giladi A Lara-Astiaso D. The spectrum and regulatory landscape of intestinal innate lymphoid cells are shaped by the microbiome. Cell. (2016) 166:1231–46.e13. 10.1016/j.cell.2016.07.043 27545347

[40] Zhang K Xu X Pasha MA Siebel CW Costello A Haczku A. Cutting edge: notch signaling promotes the plasticity of group-2 innate lymphoid cells. J Immunol. (2017) 198:1798–803. 10.4049/jimmunol.1601421 28115527 PMC5321819

[41] Bal SM Bernink JH Nagasawa M Groot J Shikhagaie MM Golebski K. IL-1β, IL-4 and IL-12 control the fate of group 2 innate lymphoid cells in human airway inflammation in the lungs. Nat Immunol. (2016) 17:636–45. 10.1038/ni.3444 27111145

[42] Ohne Y Silver JS Thompson-Snipes L Collet MA Blanck JP Cantarel BL IL-1 is a critical regulator of group 2 innate lymphoid cell function and plasticity. Nat Immunol. (2016) 17:646–55. 10.1038/ni.3447 27111142

[43] Silver JS Kearley J Copenhaver AM Sanden C Mori M Yu L Inflammatory triggers associated with exacerbations of COPD orchestrate plasticity of group 2 innate lymphoid cells in the lungs. Nat Immunol. (2016) 17:626–35. 10.1038/ni.3443 27111143 PMC5345745

[44] Lim AI Menegatti S Bustamante J Le Bourhis L Allez M Rogge L. IL-12 drives functional plasticity of human group 2 innate lymphoid cells. J Exp Med. (2016) 213:569–83. 10.1084/jem.20151750 26976630 PMC4821648

[45] Teunissen mBM Munneke JM Bernink JH Spuls PI Res PCM Te Velde A. Composition of innate lymphoid cell subsets in the human skin: enrichment of NCR(+) ILC3 in lesional skin and blood of psoriasis patients. J Invest Dermatol. (2014) 134:2351–60. 10.1038/jid.2014.146 24658504

[46] Mjosberg JM Trifari S Crellin NK Peters CP van Drunen CM Piet B. Human IL-25- and IL-33-responsive type 2 innate lymphoid cells are defined by expression of CRTH2 and CD161. Nat Immunol. (2011) 12:1055–62. 10.1038/ni.2104 21909091

[47] Bernink JH Peters CP Munneke M te Velde AA Meijer SL Weijer K. Human type 1 innate lymphoid cells accumulate in inflamed mucosal tissues. Nat Immunol. (2013) 14:221–9. 10.1038/ni.2534 23334791

[48] Hughes T Becknell B McClory S Briercheck E Freud AG Zhang X. Stage 3 immature human natural killer cells found in secondary lymphoid tissue constitutively and selectively express the TH 17 cytokine interleukin-22. Blood. (2009) 113:4008–10. 10.1182/blood-2008-12-192443 19244159 PMC2673127

[49] Cella M Otero K Colonna M. Expansion of human NK-22 cells with IL-7, IL-2, and IL-1beta reveals intrinsic functional plasticity. Proc Natl Acad Sci USA. (2010) 107:10961–6. 10.1073/pnas.1005641107 20534450 PMC2890739

[50] Hughes T Becknell B Freud AG McClory S Briercheck E Yu J. Interleukin-1beta selectively expands and sustains interleukin-22+ immature human natural killer cells in secondary lymphoid tissue. Immunity. (2010) 32:803–14. 10.1016/j.immuni.2010.06.007 20620944 PMC3742307

[51] Klose CS Kiss EA Schwierzeck V Ebert K Hoyler T d'Hargues Y. A T-bet gradient controls the fate and function of CCR6-RORgammat+ innate lymphoid cells. Nature. (2013) 494:261–5. 10.1038/nature11813 23334414

[52] Vonarbourg C Mortha A Bui VL Hernandez PP Kiss EA Hoyler T. Regulated expression of nuclear receptor RORgammat confers distinct functional fates to NK cell receptor-expressing RORgammat(+) innate lymphocytes. Immunity. (2010) 33:736–51. 10.1016/j.immuni.2010.10.017 21093318 PMC3042726

[53] Anderson GP. Endotyping asthma: new insights into key pathogenic mechanisms in a complex, heterogeneous disease. Lancet. (2008) 372:1107–19. 10.1016/S0140-6736(08)61452-X 18805339

[54] Starkey MR McKenzie AN Belz GT Hansbro PM. Pulmonary group 2 innate lymphoid cells: surprises and challenges. Mucosal Immunol. (2019) 12:299–311. 10.1038/s41385-018-0130-4 30664706 PMC6436699

[55] Fort MM Cheung J Yen D Li J Zurawski SM Lo S. IL-25 induces IL-4, IL-5, and IL-13 and Th2-associated pathologies *in vivo*. Immunity. (2001) 15:985–95. 10.1016/S1074-7613(01)00243-6 11754819

[56] Klein Wolterink RG Kleinjan A van Nimwegen M Bergen I de Bruijn M Levani Y. Pulmonary innate lymphoid cells are major producers of IL-5 and IL-13 in murine models of allergic asthma. Eur J Immunol. (2012) 42:1106–16. 10.1002/eji.201142018 22539286

[57] Kondo Y Yoshimoto T Yasuda K Futatsugi-Yumikura S Morimoto M Hayashi N. Administration of IL-33 induces airway hyperresponsiveness and goblet cell hyperplasia in the lungs in the absence of adaptive immune system. Int Immunol. (2008) 20:791–800. 10.1093/intimm/dxn037 18448455

[58] Barlow JL Bellosi A Hardman CS Drynan LF Wong SH Cruickshank JP. Innate IL-13-producing nuocytes arise during allergic lung inflammation and contribute to airways hyperreactivity. J Allergy Clin Immunol. (2012) 129:191–8 e1–4. 10.1016/j.jaci.2011.09.041 22079492

[59] Barlow JL Peel S Fox J Panova V Hardman CS Camelo A. IL-33 is more potent than IL-25 in provoking IL-13-producing nuocytes (type 2 innate lymphoid cells) and airway contraction. J Allergy Clin Immunol. (2013) 132:933–41. 10.1016/j.jaci.2013.05.012 23810766

[60] Li Y Chen S Chi Y Yang Y Chen X Wang H. Kinetics of the accumulation of group 2 innate lymphoid cells in IL-33-induced and IL-25-induced murine models of asthma: a potential role for the chemokine CXCL16. Cell Mol Immunol. (2019) 16:75–86. 10.1038/s41423-018-0182-0 30467418 PMC6318283

[61] Watarai H Sekine-Kondo E Shigeura T Motomura Y Yasuda T Satoh R. Development and function of invariant natural killer T cells producing T(h)2- and T(h)17-cytokines. PLoS Biol. (2012) 10:e1001255. 10.1371/journal.pbio.1001255 22346732 PMC3274505

[62] Walford HH Lund SJ Baum RE White AA Bergeron CM Husseman J. Increased ILC2s in the eosinophilic nasal polyp endotype are associated with corticosteroid responsiveness. Clin Immunol. (2014) 155:126–35. 10.1016/j.clim.2014.09.007 25236785 PMC4254351

[63] Fan D Wang X Wang M Wang Y Zhang L Li Y. Allergen-dependent differences in ILC2s frequencies in patients with allergic rhinitis. Allergy Asthma Immunol Res. (2016) 8:216–22. 10.4168/aair.2016.8.3.216 26922931 PMC4773209

[64] van Rijt L von Richthofen H van Ree R. Type 2 innate lymphoid cells: at the cross-roads in allergic asthma. Semin Immunopathol. (2016) 38:483–96. 10.1007/s00281-016-0556-2 26965110 PMC4896999

[65] Bartemes KR Kephart GM Fox SJ Kita H. Enhanced innate type 2 immune response in peripheral blood from patients with asthma. J Allergy Clin Immunol. (2014) 134:671–8 e4. 10.1016/j.jaci.2014.06.024 25171868 PMC4149890

[66] Smith SG Chen R Kjarsgaard M Huang C Oliveria JP O'Byrne PM. Increased numbers of activated group 2 innate lymphoid cells in the airways of patients with severe asthma and persistent airway eosinophilia. J Allergy Clin Immunol. (2016) 137:75–86 e8. 10.1016/j.jaci.2015.05.037 26194544

[67] Stadhouders R Li BWS de Bruijn MJW Gomez A Rao TN Fehling HJ. Epigenome analysis links gene regulatory elements in group 2 innate lymphocytes to asthma susceptibility. J Allergy Clin Immunol. (2018) 142:1793–807. 10.1016/j.jaci.2017.12.1006 29486229

[68] Kim HY Lee HJ Chang YJ Pichavant M Shore SA Fitzgerald KA. Interleukin-17-producing innate lymphoid cells and the NLRP3 inflammasome facilitate obesity-associated airway hyperreactivity. Nat Med. (2014) 20:54–61. 10.1038/nm.3423 24336249 PMC3912313

[69] Hekking PP Loza MJ Pavlidis S de Meulder B Lefaudeux D Baribaud F. Pathway discovery using transcriptomic profiles in adult-onset severe asthma. J Allergy Clin Immunol. (2018) 141:1280–90. 10.1016/j.jaci.2017.06.037 28756296

[70] Schnyder B Lima C Schnyder-Candrian S. Interleukin-22 is a negative regulator of the allergic response. Cytokine. (2010) 50:220–7. 10.1016/j.cyto.2010.02.003 20194033

[71] Besnard AG Sabat R Dumoutier L Renauld JC Willart M Lambrecht B. Dual Role of IL-22 in allergic airway inflammation and its cross-talk with IL-17A. Am J Respir Crit Care Med. (2011) 183:1153–63. 10.1164/rccm.201008-1383OC 21297073

[72] Taube C Tertilt C Gyulveszi G Dehzad N Kreymborg K Schneeweiss K. IL-22 is produced by innate lymphoid cells and limits inflammation in allergic airway disease. PLoS ONE. (2011) 6:e21799. 10.1371/journal.pone.0021799 21789181 PMC3138740

[73] Gray J Oehrle K Worthen G Alenghat T Whitsett J Deshmukh H. Intestinal commensal bacteria mediate lung mucosal immunity and promote resistance of newborn mice to infection. Sci Transl Med. (2017) 9:eaaf9412. 10.1126/scitranslmed.aaf9412 28179507 PMC5880204

[74] Liao B Cao PP Zeng M Zhen Z Wang H Zhang YN. Interaction of thymic stromal lymphopoietin, IL-33, and their receptors in epithelial cells in eosinophilic chronic rhinosinusitis with nasal polyps. Allergy. (2015) 70:1169–80. 10.1111/all.12667 26095319

[75] Shaw JL Fakhri S Citardi MJ Porter PC Corry DB Kheradmand F. IL-33-responsive innate lymphoid cells are an important source of IL-13 in chronic rhinosinusitis with nasal polyps. Am J Respir Crit Care Med. (2013) 188:432–9. 10.1164/rccm.201212-2227OC 23805875 PMC5448506

[76] Miljkovic D Bassiouni A Cooksley C Ou J Hauben E Wormald PJ. Association between group 2 innate lymphoid cells enrichment, nasal polyps and allergy in chronic rhinosinusitis. Allergy. (2014) 69:1154–61. 10.1111/all.12440 24924975

[77] Ho J Bailey M Zaunders J Mrad N Sacks R Sewell W Group 2 innate lymphoid cells (ILC2s) are increased in chronic rhinosinusitis with nasal polyps or eosinophilia. Clin Exp Allergy. (2015) 45:394–403. 10.1111/cea.12462 25429730

[78] Poposki JA Klingler AI Tan BK Soroosh P Banie H Lewis G. Group 2 innate lymphoid cells are elevated and activated in chronic rhinosinusitis with nasal polyps. Immun Inflamm Dis. (2017) 5:233–43. 10.1002/iid3.161 28474861 PMC5569375

[79] Ebbo M Crinier A Vely F Vivier E. Innate lymphoid cells: major players in inflammatory diseases. Nat Rev Immunol. (2017) 17:665–78. 10.1038/nri.2017.86 28804130

[80] Suzuki M Sze MA Campbell JD Brothers JF 2nd Lenburg ME McDonough JE. The cellular and molecular determinants of emphysematous destruction in COPD. Sci Rep. (2017) 7:9562. 10.1038/s41598-017-10126-2 28842670 PMC5573394

[81] Donovan C Starkey MR Kim RY Rana BMJ Barlow JL Jones B. Roles for T/B lymphocytes and ILC2s in experimental chronic obstructive pulmonary disease. J Leukoc Biol. (2019) 105:143–50. 10.1002/JLB.3AB0518-178R 30260499 PMC6487813

[82] Monticelli LA Buck MD Flamar AL Saenz SA Tait Wojno ED Yudanin NA. Arginase 1 is an innate lymphoid-cell-intrinsic metabolic checkpoint controlling type 2 inflammation. Nat Immunol. (2016) 17:656–65. 10.1038/ni.3421 27043409 PMC4873382

[83] Di Stefano A Caramori G Gnemmi I Contoli M Vicari C Capelli A. T helper type 17-related cytokine expression is increased in the bronchial mucosa of stable chronic obstructive pulmonary disease patients. Clin Exp Immunol. (2009) 157:316–24. 10.1111/j.1365-2249.2009.03965.x 19604272 PMC2730858

[84] Shikhagaie MM Bjorklund AK Mjosberg J Erjefalt JS Cornelissen AS Ros XR. Neuropilin-1 is expressed on lymphoid tissue residing LTi-like group 3 innate lymphoid cells and associated with ectopic lymphoid aggregates. Cell Rep. (2017) 18:1761–73. 10.1016/j.celrep.2017.01.063 28199847 PMC5318658

[85] Prieto A Reyes E Bernstein ED Martinez B Monserrat J Izquierdo JL. Defective natural killer and phagocytic activities in chronic obstructive pulmonary disease are restored by glycophosphopeptical (inmunoferon). Am J Respir Crit Care Med. (2001) 163:1578–83. 10.1164/ajrccm.163.7.2002015 11401877

[86] Urbanowicz RA Lamb JR Todd I Corne JM Fairclough LC. Altered effector function of peripheral cytotoxic cells in COPD. Respir Res. (2009) 10:53. 10.1186/1465-9921-10-53 19545425 PMC2705911

[87] Urbanowicz RA Lamb JR Todd I Corne JM Fairclough LC. Enhanced effector function of cytotoxic cells in the induced sputum of COPD patients. Respir Res. (2010) 11:76. 10.1186/1465-9921-11-76 20540777 PMC2891678

[88] Freeman CM Stolberg VR Crudgington S Martinez FJ Han MK Chensue SW. Human CD56+ cytotoxic lung lymphocytes kill autologous lung cells in chronic obstructive pulmonary disease. PLoS ONE. (2014) 9:e103840. 10.1371/journal.pone.0103840 25078269 PMC4117545

[89] Wortham BW Eppert BL Motz GT Flury JL Orozco-Levi M Hoebe K. NKG2D mediates NK cell hyperresponsiveness and influenza-induced pathologies in a mouse model of chronic obstructive pulmonary disease. J Immunol. (2012) 188:4468–75. 10.4049/jimmunol.1102643 22467655 PMC3331972

[90] Stolberg VR Martin B Mancuso P Olszewski MA Freeman CM Curtis JL. Role of CC chemokine receptor 4 in natural killer cell activation during acute cigarette smoke exposure. Am J Pathol. (2014) 184:454–63. 10.1016/j.ajpath.2013.10.017 24333113 PMC3906485

[91] Motz GT Eppert BL Wortham BW Amos-Kroohs RM Flury JL Wesselkamper SC. Chronic cigarette smoke exposure primes NK cell activation in a mouse model of chronic obstructive pulmonary disease. J Immunol. (2010) 184:4460–9. 10.4049/jimmunol.0903654 20228194

[92] Kearley J Silver JS Sanden C Liu Z Berlin AA White N. Cigarette smoke silences innate lymphoid cell function and facilitates an exacerbated type I interleukin-33-dependent response to infection. Immunity. (2015) 42:566–79. 10.1016/j.immuni.2015.02.011 25786179

[93] Borchers MT Wesselkamper SC Curull V Ramirez-Sarmiento A Sanchez-Font A Garcia-Aymerich J. Sustained CTL activation by murine pulmonary epithelial cells promotes the development of COPD-like disease. J Clin Invest. (2009) 119:636–49. 10.1172/JCI34462 19197141 PMC2648699

[94] Borchers MT Harris NL Wesselkamper SC Vitucci M Cosman D. NKG2D ligands are expressed on stressed human airway epithelial cells. Am J Physiol Lung Cell Mol Physiol. (2006) 291:L222–31. 10.1152/ajplung.00327.2005 16473864

[95] Jackson DJ Makrinioti H Rana BM Shamji BW Trujillo-Torralbo MB Footitt J. IL-33-dependent type 2 inflammation during rhinovirus-induced asthma exacerbations *in vivo*. Am J Respir Crit Care Med. (2014) 190:1373–82. 10.1164/rccm.201406-1039OC 25350863 PMC4299647

[96] Cayrol C Girard JP. IL-33: an alarmin cytokine with crucial roles in innate immunity, inflammation and allergy. Curr Opin Immunol. (2014) 31:31–7. 10.1016/j.coi.2014.09.004 25278425

[97] Chang YJ Kim HY Albacker LA Baumgarth N McKenzie AN Smith DE. Innate lymphoid cells mediate influenza-induced airway hyper-reactivity independently of adaptive immunity. Nat Immunol. (2011) 12:631–8. 10.1038/ni.2045 21623379 PMC3417123

[98] C.Bauer MT Morissette MC Stampfli MR The influence of cigarette smoking on viral infections: translating bench science to impact COPD pathogenesis and acute exacerbations of COPD clinically. Chest. (2013) 143:196–206. 10.1378/chest.12-0930 23276842

[99] Vashist N Trittel S Ebensen T Chambers BJ Guzman CA Riese P. Influenza-activated ILC1s contribute to antiviral immunity partially influenced by differential GITR expression. Front Immunol. (2018) 9:505. 10.3389/fimmu.2018.00505 29623077 PMC5874297

[100] Wynn TA. Integrating mechanisms of pulmonary fibrosis. J Exp Med. (2011) 208:1339–50. 10.1084/jem.20110551 21727191 PMC3136685

[101] Hams E Armstrong ME Barlow JL Saunders SP Schwartz C Cooke G. IL-25 and type 2 innate lymphoid cells induce pulmonary fibrosis. Proc Natl Acad Sci USA. (2014) 111:367–72. 10.1073/pnas.1315854111 24344271 PMC3890791

[102] Bonniaud P Martin G Margetts PJ Ask K Robertson J Gauldie J. Connective tissue growth factor is crucial to inducing a profibrotic environment in “fibrosis-resistant” BALB/c mouse lungs. Am J Respir Cell Mol Biol. (2004) 31:510–6. 10.1165/rcmb.2004-0158OC 15256388

[103] Marvie P Lisbonne M L'Helgoualc'h A Rauch M Turlin B Preisser L. Interleukin-33 overexpression is associated with liver fibrosis in mice and humans. J Cell Mol Med. (2010) 14:1726–39. 10.1111/j.1582-4934.2009.00801.x 19508382 PMC3829034

[104] Jiang D Liang J Hodge J Lu B Zhu Z Yu S. Regulation of pulmonary fibrosis by chemokine receptor CXCR3. J Clin Invest. (2004) 114:291–9. 10.1172/JCI16861 15254596 PMC449741

[105] Khalil N Parekh TV O'Connor R Antman N Kepron W Yehaulaeshet T. Regulation of the effects of TGF-beta 1 by activation of latent TGF-beta 1 and differential expression of TGF-beta receptors (T beta R-I and T beta R-II) in idiopathic pulmonary fibrosis. Thorax. (2001) 56:907–15. 10.1136/thorax.56.12.907 11713352 PMC1745982

[106] Aquino-Galvez A Perez-Rodriguez M Camarena A Falfan-Valencia R Ruiz V Montano M. MICA polymorphisms and decreased expression of the MICA receptor NKG2D contribute to idiopathic pulmonary fibrosis susceptibility. Hum Genet. (2009) 125:639–48. 10.1007/s00439-009-0666-1 19363685

[107] Trottein F Paget C. Natural killer T cells and mucosal-associated invariant T cells in lung infections. Front Immunol. (2018) 9:1750. 10.3389/fimmu.2018.01750 30116242 PMC6082944

[108] Born WK Lahn M Takeda K Kanehiro A O'Brien RL Gelfand EW. Role of gammadelta T cells in protecting normal airway function. Respir Res. (2000) 1:151–8. 10.1186/rr26 11667979 PMC59553

[109] Kinjo Y Wu D Kim G Xing GW Poles MA Ho DD. Recognition of bacterial glycosphingolipids by natural killer T cells. Nature. (2005) 434:520–5. 10.1038/nature03407 15791257

[110] Mattner J Debord KL Ismail N Goff RD Cantu C 3rd Zhou D. Exogenous and endogenous glycolipid antigens activate NKT cells during microbial infections. Nature. (2005) 434:525–9. 10.1038/nature03408 15791258

[111] Le Bourhis L Martin E Peguillet I Guihot A Froux N Core M. Antimicrobial activity of mucosal-associated invariant T cells. Nat Immunol. (2010) 11:701–8. 10.1038/ni.1890 20581831

[112] Bonneville M O'Brien RL Born WK. Gammadelta T cell effector functions: a blend of innate programming and acquired plasticity. Nat Rev Immunol. (2010) 10:467–78. 10.1038/nri2781 20539306

[113] Martin B Hirota K Cua DJ Stockinger B Veldhoen M. Interleukin-17-producing gammadelta T cells selectively expand in response to pathogen products and environmental signals. Immunity. (2009) 31:321–30. 10.1016/j.immuni.2009.06.020 19682928

[114] Umetsu DT Dekruyff RH. Natural killer T cells are important in the pathogenesis of asthma: the many pathways to asthma. J Allergy Clin Immunol. (2010) 125:975–9. 10.1016/j.jaci.2010.02.006 20338622 PMC2913488

[115] Akbari O Faul JL Hoyte EG Berry GJ Wahlstrom J Kronenberg M. CD4+ invariant T-cell-receptor+ natural killer T cells in bronchial asthma. N Engl J Med. (2006) 354:1117–29. 10.1056/NEJMoa053614 16540612

[116] Koh YI Shim JU Wi JO Han ER Jin NC Oh SH. Inverse association of peripheral blood CD4(+) invariant natural killer T cells with atopy in human asthma. Hum Immunol. (2010) 71:186–91. 10.1016/j.humimm.2009.10.011 19879910

[117] Vijayanand P Seumois G Pickard C Powell RM Angco G Sammut D. Invariant natural killer T cells in asthma and chronic obstructive pulmonary disease. N Engl J Med. (2007) 356:1410–22. 10.1056/NEJMoa064691 17409322

[118] Akbari O Stock P Meyer E Kronenberg M Sidobre S Nakayama T. Essential role of NKT cells producing IL-4 and IL-13 in the development of allergen-induced airway hyperreactivity. Nat Med. (2003) 9:582–8. 10.1038/nm851 12669034

[119] Chi SY Ban HJ Kwon YS Oh IJ Kim KS Kim YI. Invariant natural killer T cells in chronic obstructive pulmonary disease. Respirology. (2012) 17:486–92. 10.1111/j.1440-1843.2011.02104.x 22098381

[120] Forsslund Mikko M Karimi R Grunewald J Wheelock AM Wahlstrom J Skold CM. Distribution of T-cell subsets in BAL fluid of patients with mild to moderate COPD depends on current smoking status and not airway obstruction. Chest (2014) 145:711–22. 10.1378/chest.13-0873 24264182

[121] Eriksson Strom J Pourazar J Linder R Blomberg A Lindberg A Bucht A. Cytotoxic lymphocytes in COPD airways: increased NK cells associated with disease, iNKT and NKT-like cells with current smoking. Respir Res. (2018) 19:244. 10.1186/s12931-018-0940-7 30526599 PMC6286566

[122] Kim EY Battaile JT Patel AC You Y Agapov E Grayson MH. Persistent activation of an innate immune response translates respiratory viral infection into chronic lung disease. Nat Med. (2008) 14:633–40. 10.1038/nm1770 18488036 PMC2575848

[123] Kim HY Chang YJ Subramanian S Lee HH Albacker LA Matangkasombut P. Innate lymphoid cells responding to IL-33 mediate airway hyperreactivity independently of adaptive immunity. J Allergy Clin Immunol. (2012) 129:216–27 e1–6. 10.1016/j.jaci.2011.10.036 22119406 PMC3246069

[124] Dusseaux M Martin E Serriari N Peguillet I Premel V Louis D. Human MAIT cells are xenobiotic-resistant, tissue-targeted, CD161hi IL-17-secreting T cells. Blood. (2011) 117:1250–9. 10.1182/blood-2010-08-303339 21084709

[125] Godfrey DI Uldrich AP McCluskey J Rossjohn J Moody DB. The burgeoning family of unconventional T cells. Nat Immunol. (2015) 16:1114–23. 10.1038/ni.3298 26482978

[126] Rahimpour A Koay HF Enders A Clanchy R Eckle SB Meehan B. Identification of phenotypically and functionally heterogeneous mouse mucosal-associated invariant T cells using MR1 tetramers. J Exp Med. (2015) 212:1095–108. 10.1084/jem.20142110 26101265 PMC4493408

[127] Hinks TS Wallington JC Williams AP Djukanovic R Staples KJ Wilkinson TM. Steroid-induced deficiency of mucosal-associated invariant T cells in the chronic obstructive pulmonary disease lung. Implications for nontypeable haemophilus influenzae infection. Am J Respir Crit Care Med. (2016) 194:1208–18. 10.1164/rccm.201601-0002OC 27115408 PMC5114442

[128] Kwon YS Jin HM Cho YN Kim MJ Kang JH Jung HJ. Mucosal-Associated invariant T cell deficiency in chronic obstructive pulmonary disease. COPD. (2016) 13:196–202. 10.3109/15412555.2015.1069806 26552490

[129] Hinks TS Zhou X Staples KJ Dimitrov BD Manta A Petrossian T. Innate and adaptive T cells in asthmatic patients: Relationship to severity and disease mechanisms. J Allergy Clin Immunol. (2015) 136:323–33. 10.1016/j.jaci.2015.01.014 25746968 PMC4534770

[130] Ishimori A Harada N Chiba A Harada S Matsuno K Makino F. Circulating activated innate lymphoid cells and mucosal-associated invariant T cells are associated with airflow limitation in patients with asthma. Allergol Int. (2017) 66:302–9. 10.1016/j.alit.2016.07.005 27575652

[131] Cheng M Hu S. Lung-resident gammadelta T cells and their roles in lung diseases. Immunology. (2017) 151:375–84. 10.1111/imm.12764 28555812 PMC5506441

[132] Simonian PL Roark CL Wehrmann F Lanham AM Born WK O'Brien RL. IL-17A-expressing T cells are essential for bacterial clearance in a murine model of hypersensitivity pneumonitis. J Immunol. (2009) 182:6540–9. 10.4049/jimmunol.0900013 19414809 PMC2766088

[133] Hodge G Mukaro V Holmes M Reynolds PN Hodge S. Enhanced cytotoxic function of natural killer and natural killer T-like cells associated with decreased CD94 (Kp43) in the chronic obstructive pulmonary disease airway. Respirology. (2013) 18:369–76. 10.1111/j.1440-1843.2012.02287.x 23062183

[134] Pichavant M Remy G Bekaert S Le Rouzic O Kervoaze G Vilain E. Oxidative stress-mediated iNKT-cell activation is involved in COPD pathogenesis. Mucosal Immunol. (2014) 7:568–78. 10.1038/mi.2013.75 24172846 PMC3998637

[135] Wang J Urbanowicz RA Tighe PJ Todd I Corne JM Fairclough LC. Differential activation of killer cells in the circulation and the lung: a study of current smoking status and chronic obstructive pulmonary disease (COPD). PLoS ONE. (2013) 8:e58556. 10.1371/journal.pone.0058556 23505535 PMC3594304

[136] Ekberg-Jansson A Andersson B Avra E Nilsson O Lofdahl CG. The expression of lymphocyte surface antigens in bronchial biopsies, bronchoalveolar lavage cells and blood cells in healthy smoking and never-smoking men, 60 years old. Respir Med. (2000) 94:264–72. 10.1053/rmed.1999.0735 10783938

[137] Majo J Ghezzo H Cosio MG. Lymphocyte population and apoptosis in the lungs of smokers and their relation to emphysema. Eur Respir J. (2001) 17:946–53. 10.1183/09031936.01.17509460 11488331

[138] Richmond I Pritchard GE Ashcroft T Corris PA Walters EH. Distribution of gamma delta T-cells in the bronchial tree of smokers and non-smokers. J Clin Pathol. (1993) 46:926–30. 10.1136/jcp.46.10.926 8227410 PMC501620

[139] Roos-Engstrand E Ekstrand-Hammarstrom B Pourazar J Behndig AF Bucht A Blomberg A. Influence of smoking cessation on airway T lymphocyte subsets in COPD. COPD. (2009) 6:112–20. 10.1080/15412550902755358 19378224

[140] Pons J Sauleda J Ferrer JM Barcelo B Fuster A Regueiro V. Blunted gamma delta T-lymphocyte response in chronic obstructive pulmonary disease. Eur Respir J. (2005) 25:441–6. 10.1183/09031936.05.00069304 15738286

[141] Urboniene D Babusyte A Lotvall J Sakalauskas R Sitkauskiene B. Distribution of gammadelta and other T-lymphocyte subsets in patients with chronic obstructive pulmonary disease and asthma. Respir Med. (2013) 107:413–23. 10.1016/j.rmed.2012.11.012 23273406

[142] Vanaudenaerde BM Verleden SE Vos R De Vleeschauwer SI Willems-Widyastuti A Geenens R. Innate and adaptive interleukin-17-producing lymphocytes in chronic inflammatory lung disorders. Am J Respir Crit Care Med. (2011) 183:977–86. 10.1164/rccm.201007-1196PP 21097694

[143] Bayes HK Ritchie ND Evans TJ. Interleukin-17 is required for control of chronic lung infection caused by pseudomonas aeruginosa. Infect Immun. (2016) 84:3507–16. 10.1128/IAI.00717-16 27698020 PMC5116727

[144] Bozinovski S Seow HJ Chan SP Anthony D McQualter J Hansen M. Innate cellular sources of interleukin-17A regulate macrophage accumulation in cigarette- smoke-induced lung inflammation in mice. Clin Sci (Lond). (2015) 129:785–96. 10.1042/CS20140703 26201093 PMC4613531

[145] Pichavant M Sharan R Le Rouzic O Olivier C Hennegrave F Remy G. IL-22 defect during streptococcus pneumoniae infection triggers exacerbation of chronic obstructive pulmonary disease. EBioMedicine. (2015) 2:1686–96. 10.1016/j.ebiom.2015.09.040 26870795 PMC4740310

[146] Hong MJ Gu BH Madison MC Landers C Tung HY Kim M. Protective role of gammadelta T cells in cigarette smoke and influenza infection. Mucosal Immunol. (2018) 11:894–908. 10.1038/mi.2017.93 29091081 PMC5930147

[147] Vivier E van de Pavert SA Cooper MD Belz GT. The evolution of innate lymphoid cells. Nat Immunol. (2016) 17:790–4. 10.1038/ni.3459 27328009 PMC5287353

